# Surgical Timing and Outcomes in Esophageal Cancer: Insights from One- and Two-Stage Esophagectomies in a Polish Cohort

**DOI:** 10.3390/jcm14124301

**Published:** 2025-06-17

**Authors:** Bartłomiej Strzelec, Piotr Paweł Chmielewski, Wojciech Kielan, Julia Rudno-Rudzińska

**Affiliations:** 1 University Center of General and Oncologic Surgery, Wroclaw Medical University, 50-556 Wrocław, Poland; bstrzelec94@interia.pl (B.S.); wojciech.kielan@umw.edu.pl (W.K.);; 2 Division of Anatomy, Department of Human Morphology and Embryology, Faculty of Medicine, Wroclaw Medical University, 6a Chalubinskiego Street, 50-368 Wrocław, Poland

**Keywords:** esophageal cancer, esophagectomy, postoperative complications, anastomotic leakage, neoadjuvant therapy, one-stage esophagectomy, two-stage esophagectomy, surgical outcomes, Ivor Lewis procedure

## Abstract

**Objectives**: Esophagectomy is a central component of surgical treatment for esophageal cancer, with both one- and two-stage procedures frequently employed. However, these procedures are associated with a high rate of postoperative complications. This study aimed to assess the rates and types of complications following one- and two-stage esophagectomies, and to identify predictors of adverse outcomes in patients with esophageal cancer. **Methods**: We analyzed clinical data from patients undergoing one-stage (Ivor Lewis) or two-stage esophagectomies. Postoperative complications were defined as events occurring within 30 days after surgery. Variables such as patient demographics, clinical staging, histological tumor grade, and neoadjuvant chemoradiotherapy were assessed for their association with complications. Statistical analyses included logistic regression and chi-squared tests. **Results**: Among 61 patients, postoperative complications occurred in 24.6% of cases. The most frequent were pneumonia (22.2%), anastomotic leakage (22.2%), and hemothorax (27.8%). Significant predictors of complications included intraoperative disease staging, histological tumor grade, and the use of neoadjuvant chemoradiotherapy. The odds ratio for complications following neoadjuvant chemoradiotherapy was 8.75. The frequency of anastomotic leakage was similar in one- and two-stage procedures (30.8% vs. 26.3%, respectively). **Conclusions**: Postoperative complications remain a significant challenge in esophageal cancer surgery, particularly in the context of advanced disease or neoadjuvant chemoradiotherapy. These findings underscore the necessity for precise surgical planning and comprehensive postoperative care to mitigate risks and optimize patient outcomes. While postoperative risk is high, it is primarily driven by tumor characteristics and preoperative therapy.

## 1. Introduction

Esophageal cancer (EC) is a highly lethal malignancy that represents a major global health burden, with approximately 500,000–600,000 new cases and 500,000 deaths annually despite its relatively low incidence [1,2,3,4,5]. Patients typically present with dysphagia, weight loss, and cachexia, reflecting the disease’s rapid progression and poor prognosis [3]. Despite advances in multimodal therapy, including immunotherapy and targeted approaches, five-year survival remains below 20% in most cohorts [4,5,6]. Diagnosis typically involves high-resolution endoscopic ultrasonography and computed tomography (CT), with histopathological staging guiding treatment decisions. The advanced age at presentation, combined with EC’s aggressive biology, places considerable strain on health systems and underscores the urgent need for novel therapies targeting its molecular drivers and strategies to improve early detection.

Two predominant subtypes of EC are squamous cell carcinoma (SCC) and adenocarcinoma (AC), which differ markedly in epidemiology, pathophysiology, and clinical presentation. SCC is strongly associated with heavy tobacco and alcohol use, whereas AC is more commonly linked to obesity and chronic gastroesophageal reflux disease (GERD) [3,7]. SCC accounts for approximately 90% of cases worldwide, with a higher prevalence in regions such as Asia, East Africa, and South America [8], while AC predominates in Western countries [9]. Both subtypes have identifiable precursor lesions that, if detected early, are amenable to endoscopic ablative therapies. Nonetheless, the majority of cases present at locally advanced or metastatic stages, requiring complex, multimodal treatment approaches. Effective management of EC typically combines chemotherapy, chemoradiotherapy (CRT), and surgical resection, tailored to the patient’s disease stage and functional status. For advanced or metastatic cases, palliative chemotherapy remains the cornerstone of care, with targeted agents, such as trastuzumab for HER2-positive tumors, offering survival benefits [1]. Immuno-oncology therapies, particularly checkpoint inhibitors, have also shown promising results [10,11,12]. Despite these advances, surgical resection remains the cornerstone of curative therapy, particularly when combined with induction CRT for tumors located more than 5 cm from the gastroesophageal junction. Esophagectomy is among the most complex and high-risk surgical procedures, with substantial rates of perioperative and postoperative complications [13,14,15].

Perioperative complications include hemorrhage from major vessels (e.g., the azygos vein or aorta), cardiac arrhythmias, hemodynamic instability due to compression of the great veins, and injuries to the recurrent laryngeal nerves or tracheobronchial tree. Postoperative complications range from respiratory issues, such as pneumonia and acute respiratory distress syndrome (ARDS), to wound infections, pleural effusion, chylothorax, and anastomotic leaks (AL) [16,17,18,19,20]. Late complications, such as anastomotic strictures, postprandial syndromes, gastroparesis, and chronic diarrhea, can significantly impair quality of life and long-term outcomes. Among these, AL is one of the most severe and potentially fatal events following esophagectomy or gastrointestinal continuity restoration in two-stage procedures. The incidence of AL varies widely, ranging from 2% to 20%, depending on factors such as patient age, disease stage, preoperative weight loss, tumor location, histological subtype, and overall health status [21]. AL significantly increases treatment costs, prolongs hospitalization, and elevates mortality rates, which range between 2% and 12% [22].

Gastrointestinal reconstruction after esophagectomy varies by conduit selection—such as the gastric tube, jejunum, right colon with terminal ileum, or left colon—and surgical technique, both of which strongly influence anastomotic integrity and complication risk. Reconstruction is typically delayed two to three months after resection to allow for nutritional recovery, improved pulmonary function, and overall patient stabilization before the second stage. Among postoperative complications, respiratory events, particularly pneumonia, are the most frequent and carry the greatest morbidity. Pneumonia may progress to ARDS, substantially worsening the prognosis. These pulmonary complications are closely associated with poorer patient outcomes, longer intensive care and hospital stays, increased health-care costs, and diminished long-term quality of life [19].

Given the high incidence and potentially fatal nature of postoperative complications following esophageal resection and reconstruction, identifying key risk factors and implementing preventive strategies are essential for improving surgical outcomes. This long-term study involved a homogeneous cohort composed entirely of Caucasian individuals and represents a relatively large patient population. It aims to thoroughly assess the risk factors associated with postoperative complications in EC treatment. In addition, this study provides an in-depth analysis of esophageal reconstruction techniques, with a particular emphasis on AL, offering new insights from modern cohorts to optimize surgical strategies and reduce postoperative risks in the management of EC.

## 2. Materials and Methods

### 2.1. Study Design and Data Collection

The data for this study were collected retrospectively from medical records and histopathological reports of patients treated for EC at the Department of General and Gastrointestinal Surgery and the Second Department of General and Oncological Surgery in Wroclaw, Poland, between 2008 and 2022. A total of 78 patients were initially enrolled; however, 17 were excluded due to rare esophageal tumors such as lipomas, sarcomas, and fibromas. Thus, the final analysis included 61 patients diagnosed with either AC or SCC. Ethical approval was obtained from the Ethics Committee of Wroclaw Medical University on 29 June 2023.

Patients were stratified into subgroups based on demographic, clinical, and pathological characteristics (Table 1). The analyzed variables included age, sex, tumor histology, surgical approach, preoperative CRT, resection radicality, tumor staging (pretreatment and postsurgical restaging), histological grading, and postoperative complications.

All patients underwent computed tomography (CT) of the chest, abdomen, and pelvis for initial staging, along with upper endoscopy and biopsy to determine tumor grade and histological subtype. Baseline laboratory blood tests included complete blood count with differential, bilirubin, alanine aminotransferase (ALT), aspartate aminotransferase (AST), gamma-glutamyl transpeptidase (GGT), alkaline phosphatase (ALP), lipase, amylase, total protein, albumin, activated partial thromboplastin time (APTT), and international normalized ratio (INR). Each patient also underwent a 12-lead electrocardiogram (ECG), and the complete set of test results was reviewed by the anesthesiology team to determine suitability for general anesthesia.

Informed consent for surgical intervention and, if necessary, blood product transfusion was obtained from each patient at least 24 h before the operation. This 24-h period allowed adequate time for patients to consider the proposed treatment, ask questions, and receive answers. Obtaining informed consent was deemed necessary for surgical qualification. The decision regarding the type of surgical treatment, either single-stage or two-stage esophagectomy, was made by a multidisciplinary board comprising a surgeon, an anesthesiologist, a specialist in parenteral nutrition, and a gastroenterologist. Patient preferences, laboratory findings, comorbidities, and overall health status were carefully considered to determine the most appropriate and individualized treatment strategy.

Postoperative pathological staging was assigned by specialist pathologists according to the pTNM classification. From 2014 onward, induction CRT was delivered as per established protocols: patients with tumors staged ≤ T2 received 41.3 Gy following the CROSS regimen, while those with T3–T4 lesions underwent definitive chemoradiotherapy (dCRT, 52 Gy); nodal status (N-category) did not influence eligibility for CRT. 22 patients received induction CRT and surgery, whereas 39 patients underwent surgery alone.

In the two-stage cohort, the first operation comprised esophagectomy via right posterolateral thoracotomy, midline upper laparotomy, and a cervical incision, with salivary fistula creation and feeding gastrostomy placement. Reconstruction of the gastrointestinal conduit was deferred to the second stage via laparotomy and a cervical anastomotic incision. Thirteen patients underwent a one-stage Ivor Lewis esophagectomy.

After surgical treatment, these patients were monitored according to a standard postoperative care protocol, which included alternating chest CT and gastroscopy every three months during the first two years. Positron emission tomography (PET) was performed at the one-year mark, and follow-up then alternated between gastroscopy and chest CT every six months for up to five years. Nutritional status was supported in all patients, with parenteral nutrition employed for those undergoing Ivor Lewis procedures and enteral nutrition through feeding gastrostomy or microjejunostomy for patients undergoing two-stage surgeries.

The treatment protocol remained consistent throughout the study period. No significant modifications were made to the qualification process or the surgical management of patients. The only major change was the introduction of neoadjuvant CRT in 2014, in accordance with the European Society for Medical Oncology (ESMO) guidelines for the treatment of EC. Maintaining this consistent protocol ensured continuity and uniformity in patient care, thereby minimizing potential confounding factors and enhancing the reliability of the findings.

### 2.2. Statistical Analyses

The analysis was performed using Statistica for Windows, version 13.1 (StatSoft, Tulsa, OK, USA). Continuous variables were described using means ± standard deviations (SDs) and medians with interquartile ranges (*Q*1–*Q*3). The normality of data distribution was assessed using a goodness-of-fit test for normality. For comparisons between two independent groups, Student’s *t*-test was used for normally distributed variables, while the Mann–Whitney *U* test was applied for non-normally distributed variables. Associations between categorical variables were evaluated using the chi-squared test of independence. Logistic regression models were used to assess the effect of multiple variables, including their interactions, on binary outcomes. Statistical significance was defined as *p* ≤ 0.05.

## 3. Results

Postoperative complications were observed in 15 patients (24.6%), accounting for 18 distinct events. Complications were defined as adverse events occurring within 30 days of surgery with a direct causal relationship to the procedure. In three patients, two complications occurred concurrently. The observed complications included pneumonia (*N* = 4; 22.2%), delirium (*N* = 1; 5.6%), hemothorax (*N* = 5; 27.8%), pyloric mucosal bleeding following pyloroplasty (*N* = 1; 5.6%), pneumothorax (*N* = 2; 11.1%), postoperative wound hematoma and infection (*N* = 1; 5.6%), and AL (*N* = 4; 22.2%) (Table 2).

No statistically significant correlations were identified between the occurrence of postoperative complications and variables such as tumor histological type (*p* = 0.681), sex (*p* = 0.752), surgical method (*p* = 0.826), resection radicality (*p* = 0.836), age (*p* = 0.469), or clinical stage before treatment (*p* = 0.948) (Table 3). However, statistically significant associations were found between the occurrence of complications and the intraoperative clinical stage (*p* = 0.004), histological grade (*p* = 0.002), disease progression (*p* = 0.001), use of induction CRT (*p* = 0.002), and the absence of residual tumor cells in histological specimens (*p* = 0.001). The odds ratio (OR) for postoperative complications associated with induction CRT was 8.75.

A second-stage surgical procedure was performed in 38 patients (79.2%). In most cases, reconstruction utilized the distal ileum and the right part of the colon in an isoperistaltic configuration, based on the middle colic or middle and right colic arteries (*N* = 26; 68.4%). Other reconstruction approaches included using the right colon in an isoperistaltic arrangement based on the middle and predominantly right colic arteries (*N* = 8; 21.1%), the left colon in an antiperistaltic arrangement based on the middle colic artery (*N* = 1; 2.6%), and the jejunum (*N* = 3; 7.9%).

The mean interval between resection and reconstruction was 14.1 weeks. Reconstruction of the esophagus was not performed in 10 patients (20.8%). The most common reasons were disease progression (*N* = 4; 8.3%), the patient’s demise (*N* = 2; 4.2%), or patient refusal of further surgery (*N* = 1; 2.1%). We performed esophageal reconstruction using the conventional approach, namely a cervical incision combined with an upper midline laparotomy. The conduit was placed behind the sternum.

In cases involving the use of the terminal segment of the ileum and the right colon, AL occurred in six patients (15.8%), all at the cervical anastomosis, and was successfully managed with conservative treatment. When the right colon alone was used, three cases (7.9%) of AL were observed, with two leaks occurring at the cervical anastomosis and one at the cologastric anastomosis. Additionally, one case (2.6%) of AL was reported following reconstruction using the jejunum. This leakage occurred at the cervical anastomosis and was treated conservatively with favorable outcomes.

Regardless of the reconstruction technique, AL was significantly more frequent at the cervical anastomosis (nine cases; 90% of all leaks) than at the cologastric anastomosis (one case; 10% of all leaks). Leakage at the cervical anastomosis was observed in 23.7% of all operated patients, while leakage at the lower anastomosis occurred in only 2.6% of cases (Table 4).

For single-stage esophageal reconstructions (Ivor Lewis procedure), AL was reported in four patients (30.8%), with one case (7.7%) resulting in death. The rate of AL in single-stage procedures was comparable to that in two-stage procedures (namely 30.8% vs. 26.3%). In the second stage of the two-stage procedures, no other postoperative complications were identified aside from AL.

## 4. Discussion

The primary objective of this study was to evaluate the incidence and types of complications following single-stage and two-stage esophagectomies, as well as to identify predictors of adverse outcomes in patients with EC. It is noteworthy that our findings are based on a relatively large and representative cohort—one of the largest in Poland by European standards. Given the low incidence of EC in Poland and the relatively small proportion of patients eligible for curative-intent surgery, our results constitute a valuable contribution to the existing body of evidence on the surgical management of EC within the Polish healthcare context.

The management of locally advanced EC is undergoing a paradigm shift toward personalized medicine, which integrates tumor biology with individualized treatment strategies to optimize clinical outcomes [10]. This approach recognizes the complexity of this disease in terms of histological subtypes, molecular profiles, and patient-specific factors such as age, comorbidities, and functional status. Consequently, treatment decisions should be guided by a comprehensive assessment of surgical risk, balancing the potential benefits of curative intent against the substantial impact of perioperative and postoperative complications on quality of life [23].

Personalized medicine aims to achieve maximal therapeutic benefit while minimizing treatment-related morbidity, which is particularly important given the complexity of EC management [24]. Locally advanced EC presents unique challenges due to its aggressive clinical behavior, frequent late-stage diagnosis, and the necessity for urgent, multimodal therapy, including CRT, surgery, and, increasingly, targeted immuno-oncological treatments [11,12]. Despite advances in diagnostic and therapeutic techniques, treatment-related complications remain a significant obstacle for achieving optimal outcomes [13,14,15].

Among the most severe perioperative and postoperative complications are AL, respiratory and cardiac complications, and chylothorax [25,26]. Preoperative CRT, as demonstrated in our study, was associated with an increased risk of postoperative complications. Despite this, the absence of tumor cells in postoperative specimens and favorable pathological staging underscore the prognostic benefits of preoperative CRT, which significantly improves the likelihood of achieving a radical resection and extends five-year survival. These findings support the continued use of induction CRT as a standard approach in EC management [27]. In cases of AL, endoscopic placement of covered stents, combined with targeted antibiotic therapy, nil per os (NPO), parenteral nutrition, and pleural drainage, has proven effective [28,29]. In addition, endoscopic vacuum therapy (EVT) is a rapidly evolving technique that appears to be an effective and minimally invasive option for the management of AL [29].

This approach led to full recovery in 75% of cases, with no long-term anastomotic narrowing or complications. However, in one case (25%), leakage progressed to mediastinitis and resulted in death. A standardized diagnostic protocol for AL is yet to be established. Nevertheless, a multidisciplinary approach involving risk factor identification, clinical assessment, laboratory diagnostics, imaging, and endoscopy can expedite diagnosis [30]. Early intervention may enhance long-term outcomes, shorten hospital stays, and reduce treatment costs.

As for respiratory complications, the incidence of pneumonia, which can progress to ARDS, ranges from 1.5% to 38.9%, with most reports citing an occurrence in approximately one-third of surgical patients [31]. In this study, the relatively low rate of respiratory complications compared with the literature may be attributed to early respiratory rehabilitation, enteral nutrition, and epidural analgesia. Given the severity and potential consequences of respiratory complications, these measures should form the standard of care for EC treatment.

Of the patients included in this study, 13 (21.3%) underwent single-stage esophageal reconstruction (Ivor Lewis procedure). The relatively small proportion reflects the exclusion of many patients due to prior gastrectomy or tumors in the upper third of the esophagus. For patients with significant comorbidities or high risk of complications, a two-stage procedure might offer certain advantages. The shorter initial resection reduces the risk of overlapping complications and allows optimization of the patient’s condition before reconstruction.

Furthermore, the interval between stages permits vascular regeneration at the esophageal stump, thereby improving anastomotic healing and reducing the risk of leakage. Nevertheless, in all cases where a patient is eligible for a single-stage procedure, this approach should be preferred. This benefit is supported by a prospective study demonstrating increased levels of proangiogenic factors such as vascular endothelial growth factor-A (VEGF-A) and transforming growth factor-beta (TGF-β) in the esophageal stump, which enhanced granulation tissue formation and anastomotic healing [32]. Thus, two-stage procedures may be particularly advantageous for older, frail, or comorbid patients [33].

**Figure 1:**
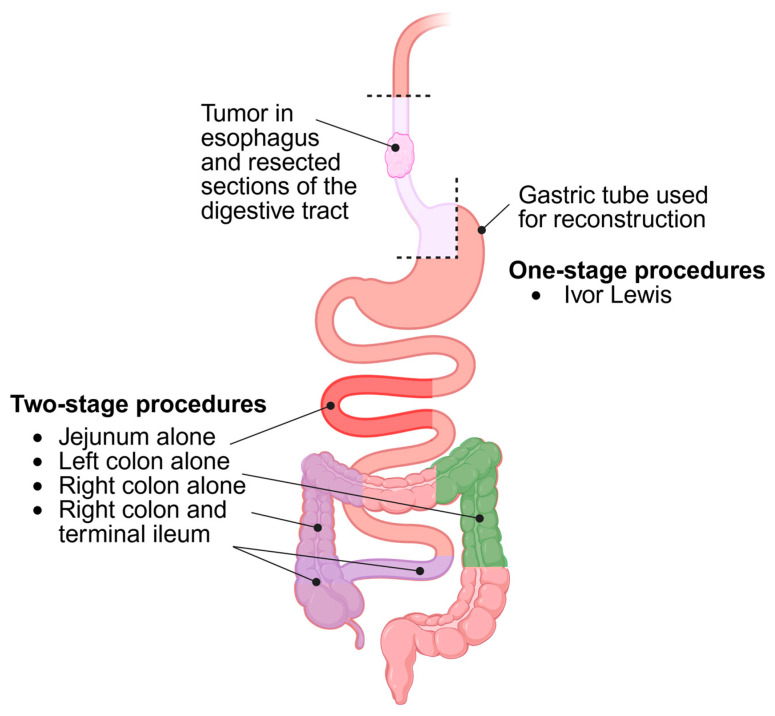
Esophageal reconstruction methods employed in this study.

Esophageal reconstruction was performed in 38 patients (79.2%), a rate comparable with those reported by other major centers. The predominant technique employed the terminal ileum and the right colon based on the middle colic artery, frequently supplemented by the right colic artery (68.4%). Other techniques included reconstruction using the right colon alone (21.1%), the left colon (2.6%), or the jejunum (7.9%) (Figure 1).

Conventional techniques, such as gastric pull-up and colon interposition, remain essential surgical approaches. The gastric pull-up is advantageous due to its simplicity and proximity, although it increases the risk of reflux. Colon interposition, particularly using the right or left colon, offers a longer graft length but requires meticulous vascular planning to ensure viability. Jejunal interposition provides a motile graft but involves more complex maneuvers and may be less commonly employed due to its technical demands.

In recent years, modern surgical techniques, such as minimally invasive surgery (MIS) and robotic-assisted approaches, have emerged as transformative tools [34,35]. These techniques minimize surgical stress, reduce complications, and accelerate recovery. Robotic-assisted surgery, in particular, enhances surgical precision and reduces potential human error, aligning with the goal of improving patient outcomes while maintaining graft functionality. The methods of esophageal reconstruction employed in this study are illustrated in Figure 1. The mean interval between resection and reconstruction was 14.1 weeks. The right colon, with its favorable vascular anatomy, long mesentery, and uniform contractility, facilitates the creation of a long graft capable of cervical anastomosis. This approach also reduces the risk of gastroesophageal reflux and halitosis [36,37]. In contrast, the jejunum is less suitable due to its short mesentery. Thus, the jejunum is rarely used for cervical anastomoses, although it can be used to restore continuity of the digestive tract within the chest. Accordingly, only 7.9% (*N* = 3) of reconstructions in this study were performed using the jejunum.

Overall, 23.7% (*N* = 9) of leaks occurred at cervical anastomoses, irrespective of the reconstruction method used, whereas only one leak (2.6%) was reported at the graft-to-stomach anastomosis. Thus, AL was observed in 23.7% of cervical anastomoses and 2.6% of cologastric anastomoses. Cervical leaks were managed conservatively with good outcomes, while the single cologastric leak was successfully treated with endoscopic stenting. Importantly, no deaths related to AL occurred during the reconstructive phase. Overall, these findings suggest the benefits of the two-stage approach in reducing severe complications in high-risk patients.

Reconstruction was not performed in 10 patients (20.8%) due to disease progression (*N* = 4; 8.3%), death (*N* = 2; 4.2%), or patient refusal (*N* = 1; 2.1%). Three patients did not return for follow-up after the first stage of treatment. However, the fact that almost 80% of patients underwent successful reconstruction with favorable outcomes underscores the effectiveness of the two-stage approach in selected groups of patients. Although leakage rates did not differ significantly between surgical techniques, the two-stage approach likely reduced risk in the most vulnerable patients. Moreover, aside from leakage, no complications occurred after the second stage, suggesting that proper preoperative preparation and nutritional optimization can mitigate postoperative risks [38,39,40].

While single-stage esophageal reconstructions (Ivor Lewis procedures) are generally preferred, two-stage procedures might offer tangible benefits for patients with significant comorbidities or high perioperative risk. By reducing operative time during resection and minimizing overlapping complications, this approach represents an important alternative for carefully selected patients. Future research should focus on the integration of advanced surgical techniques, optimization of perioperative care, and the application of molecular biology insights to enhance survival rates and quality of life for patients with esophageal cancer.

This study has certain limitations, including a modest sample size and the inability to perform multivariable analysis. However, the cohort represents a homogeneous Polish population observed over an extended period, which enhances consistency and reduces the likelihood of confounding. While the sample size may appear limited from a statistical perspective, it constitutes one of the largest and most representative cohorts available in Poland for this rare disease.

## 5. Conclusions

This study identified neoadjuvant CRT as an independent predictor of postoperative complications, highlighting its dual role in facilitating oncologic downstaging while increasing perioperative risk. Although single-stage esophagectomy, such as the Ivor Lewis procedure, remains the standard approach, two-stage operations may offer advantages in selected patients, including shorter operative times and reduced clustering of complications. Overall complication rates did not differ significantly between the two techniques. Instead, higher risks were associated with intraoperative tumor stage, histologic grade, and the use of neoadjuvant therapy. These findings underscore the importance of individualized surgical planning and coordinated, multidisciplinary postoperative care to mitigate risk and improve outcomes in patients with esophageal cancer. Nevertheless, further prospective studies are warranted to validate these results and support the standardization of the qualification process for treatment selection in this patient population.

## Figures and Tables

**Table 1:** Distribution of patients based on the analyzed characteristic.

Characteristic		Females (*N* = 19; 31.1%)	Males (*N* = 42; 68.9%)	All Patients (*N* = 61; 100%)
Age (Mean ± SD)		59.9 ± 10.3	60.5 ± 7.4	60.3 ± 8.4
Median age (Q1, Q3)		60 (57, 66)	60 (56, 66)	60 (57, 66)
Tumor histopathology	AC	0 (0%)	8 (13.1%)	8 (13.1%)
	SCC	19 (31.1%)	34 (55.7%)	53 (86.9%)
Surgical approaches	IL	5 (8.2%)	8 (13.1%)	13 (21.3%)
	TSR	14 (22.9%)	34 (55.7%)	48 (78.7%)
Preoperative chemoradiotherapy	Yes	4 (6.6%)	18 (29.5%)	22 (36.1%)
	No	15 (25.0%)	24 (39.3%)	39 (63.9%)
Radicality of resection	R0	19 (31.1%)	35 (57.4%)	54 (88.5%)
	R1	0 (0%)	7 (11.5%)	7 (11.5%)
Age subgroups (years)	<59	6 (9.8%)	16 (26.2%)	22 (36.1%)
	59–64	7 (11.5%)	11 (18.0%)	18 (29.5%)
	>64	6 (9.8%)	15 (53.4%)	21 (34.4%)
Clinical stage of the disease prior to treatment (Staging before CRT)	I	13 (21.3%)	15 (25.0%)	28 (46.0%)
	II	1 (1.6%)	10 (16.4%)	11 (18.0%)
	III	5 (8.2%)	15 (25.0%)	20 (32.8%)
	IV	0 (0%)	1 (1.6%)	1 (1.6%)
Clinical stage of the disease during surgery (Restaging, after CRT)	0	2 (3.3%)	10 (16.4%)	12 (19.7%)
	I	12 (19.7%)	10 (16.4%)	22 (36.1%)
	II	1 (1.6%)	6 (24.0%)	7 (11.5%)
	III	4 (6.6%)	14 (23.0%)	18 (30.0%)
	IV	0 (0%)	2 (3.3%)	2 (3.3%)
Cancer cells in postoperative specimens?	No	2 (3.3%)	10 (16.4%)	12 (20.0%)
	Yes	17 (27.9%)	32 (52.5%)	53 (80.0%)
Occurrence of postoperative complications?	No	15 (25.0%)	31 (50.8%)	46 (75.4%)
	Yes	4 (6.6%)	11 (18.0%)	15 (24.6%)
Histological grade of malignancy	G0	2 (3.3%)	10 (16.4%)	12 (19.7%)
	G1	4 (6.6%)	6 (9.8%)	10 (16.4%)
	G2	11 (18.0%)	19 (31.1%)	30 (49.2%)
	G3	2 (3.3%)	7 (11.5%)	9 (14.8%)

AC = adenocarcinoma; IL = Ivor Lewis; SCC = squamous cell carcinoma; TSR = two-stage resection.

**Table 2:** Type and frequency of observed postoperative complications.

Type of Complication	Number & Percentage (of All Complications)	Percentage (of All Procedures Performed)
Pneumonia	4; 22.2%	6.6%
Delirium	1; 5.6%	1.6%
Hemothorax	5; 27.8%	8.2%
Pyloric mucosal bleeding following pyloroplasty	1; 5.6%	1.6%
Pneumothorax	2; 11.1%	3.3%
Postoperative wound hematoma and infection	1; 5.6%	1.6%
Anastomotic leakage	4; 22.2%	6.6%

**Table 3:** Incidence of postoperative complications according to the examined characteristics, with results of statistical analysis.

Characteristic	Number of Patients	Number and Percentage of Patients with Complications	Chi-Squared Test, *p*-Value
Tumor histopathology	AC, *N* = 8	2; 25%	*p* = 0.681
	SCC, *N* = 53	13; 24.5%	
Sex	Females, *N* = 19	4; 21.1%	*p* = 0.752
	Males, *N* = 42	11; 26.2%	
Surgical approach	Ivor Lewis, *N* = 13	4; 30.8%	*p* = 0.826
	First stage of two-stage resection, *N* = 48	11; 22.9%	
Radicality of resection	R0, *N* = 54	13; 24.1%	*p* = 0.836
	R1, *N* = 7	2; 28.6%	
Age (years)	<59, *N* = 22	5; 22.7%	*p* = 0.469
	59–64, *N* = 18	3; 16.7%	
	>64, *N* = 21	7; 33.3%	
Clinical stage of the disease prior to treatment (Staging)	I, *N* = 29	7; 24.1%	*p* = 0.948
	II, *N* = 11	3; 27.3%	
	III, *N* = 20	5; 25%	
	IV, *N* = 1	0; 0%	
Clinical stage of the disease during surgery (Restaging)	0, *N* = 12	8; 66.7%	*p* = 0.004
	I, *N* = 22	2; 9.1%	
	II, *N* = 7	11; 14.3%	
	III, *N* = 18	4; 22.2%	
	IV, *N* = 2	0; 0%	
Grading	0, *N* = 12	8; 66.6%	*p* = 0.002
	I, *N* = 10	2; 20%	
	II, *N* = 30	4; 13.3%	
	III, *N* = 9	1; 11.1%	
Change in disease stage	Worsening, *N* = 1	0; 0%	*p* = 0.001
	No change, *N* = 40	4; 10%	
	Improvement, *N* = 20	11; 55%	
Use of preoperative chemoradiotherapy	Yes, *N* = 22	11; 50%	*p* = 0.002
	No, *N* = 39	4; 10.6%	
Presence of cancer cells in the postoperative specimen	Yes, *N* = 49	7; 14.3%	*p* = 0.001
	No, *N* = 12	8; 66.7%	

**Table 4:** Types of reconstructive procedures performed, along with the frequency and location of anastomotic leaks.

Type of Reconstruction	Number of Procedures Performed; %	Number of Anastomotic Leaks; %	Leak at the Cervical Anastomosis; %	Leak at the Lower Anastomosis; %
Using the distal ileum and right colon	26; 68.4%	6; 15.8%	6; 15.8%	0; 0%
Using the right colon alone	8; 21.1%	3; 7.9%	2; 5.3%	1; 2.6%
Using the left colon alone	1; 2.6%	0; 0%	0; 0%	0; 0%
Using the jejunum alone	3; 7.9%	1; 2.6%	1; 2.6%	0; 0%
Total	38; 100%	10; 26.3%	9; 23.7%	1; 2.6%

## Data Availability

Data are contained within the article.

## Abbreviations

The following abbreviations are used in this manuscript:

AC	Adenocarcinoma
AL	Anastomotic Leaks
ARDS	Acute Respiratory Distress Syndrome
CRT	Chemoradiotherapy
CT	Computed Tomography
EC	Esophageal Cancer
Gy	Gray
HER2	Human Epidermal Growth Factor Receptor 2
PET	Positron Emission Tomography
SCC	Squamous Cell Carcinoma
